# NG2/CSPG4 attenuates motility in mandibular fibrochondrocytes under serum starvation conditions

**DOI:** 10.3389/fcell.2023.1240920

**Published:** 2023-11-07

**Authors:** Shin Young Ahn, Mina Bagheri Varzaneh, Yan Zhao, Jacob Rozynek, Sriram Ravindran, Jonathan Banks, Minahil Chaudhry, David A. Reed

**Affiliations:** ^1^ Department of Periodontics, College of Dentistry, University of Illinois Chicago, Chicago, IL, United States; ^2^ Department of Oral Biology, College of Dentistry, University of Illinois Chicago, Chicago, IL, United States

**Keywords:** cell migration, NG2/CSPG4, chondrocytes, temporomandibular joint, collagen VI, cell adhesion, cell motility

## Abstract

The migration of mandibular fibrochondrocytes is important for the development of the mandible, the homeostasis of the mandibular cartilage, and for the capacity of the tissue to respond to injury. Mandibular fibrochondrocytes have to overcome formidable obstacles during migration including a dense and heterogeneous three-dimensional matrix. Guiding the direction of cell migration and commitment to a migratory phenotype in this microenvironment necessitates a multivalent response to chemotactic and extracellular matrix-mediated stimuli. One of the key matrix components in the cartilage of the temporomandibular joint is type VI collagen. Neuron/glial antigen 2 (NG2/CSPG4) is a transmembrane proteoglycan that binds with collagen VI and has been implicated in a wide range of cell behaviors including cell migration, motility, adhesion, and proliferation. While NG2/CSPG4 has been shown to be a key regulator of mandibular cartilage homeostasis, its role in the migration of mandibular fibrochondrocytes during normal and cell stress conditions has yet to be resolved. Here, we address this gap in knowledge by characterizing NG2/CSPG4-dependent migration in mandibular fibrochondrocytes using primary mandibular fibrochondrocytes isolated from control and full length NG2/CSPG4 knockout mice, in primary mandibular fibrochondrocytes isolated from NG2|DsRed reporter mice and in an immortalized mandibular fibrochondrocyte cell line with a mutated NG2/CSPG4 ectodomain. All three cells demonstrate similar results, with loss of the full length or truncated NG2/CSPG4 increasing the rate of cell migration in serum starvation/cell stress conditions. These findings clearly implicate NG2/CSPG4 as a key molecule in the regulation of cell migration in mandibular fibrochondrocytes in normal and cell stress conditions, underscoring the role of NG2/CSPG4 as a mechanosensitive signaling hub in the mandibular cartilage.

## 1 Introduction

The migration of chondrocytes through a dense extracellular matrix is a remarkable and understudied phenomenon in the cell and molecular biology of cartilage. Migration is critical for both development and the tissue’s response to injury. During degenerative arthropathies such as osteoarthritis (OA), the post-traumatic injury response of the cartilage is facilitated through cell migration, enabling progenitor and inflammatory cells to initiate the repair and resolution of the affected area. Migratory chondrocyte progenitor cells synthesize a fibrocartilaginous neo-matrix that improves the overall integrity of the tissue but does not perfectly recapitulate the higher-order structure and material properties of the original tissue (Seol et al., 2012; Jiang and Tuan, 2015). These progenitor cells have stem cell-like properties and can be induced to commit to a chondrogenic fate. A similar process has been reported in the cartilage of the temporomandibular joint (TMJ), with migratory cells within cartilage defects observed shortly after injury (Cledes et al., 2006). It is unknown if these migratory cells in the TMJ are the fibrocartilage stem cells recently identified from the rodent and human condyle (Robinson et al., 2015; Embree et al., 2016; Bi et al., 2020; Fan et al., 2021). Despite the mobilization of these cells, many post-natal injuries are not repaired. This is likely the result of the difficulties associated with migration through a dense and heterogeneous extracellular matrix that impairs the infiltration of repair cells into the affected area following post-traumatic injury (Morales, 2007). There is an important gap in knowledge related to the molecular mechanisms regulating the migratory potential and phenotype of chondrocytes.

When moving through a heterogeneous substrate such as cartilage, cells adaptively modify their cell surface microdomain by modifying the composition and organization of receptors, proteoglycans, cell adhesion molecules, and proteases, switching between “path finding” and “path generating” behaviors (Cattaruzza and Perris, 2005). Cells migrate through a substrate by active polymerization of the leading edge of the cell, generating cytoskeletal protrusions that adhere to the extracellular matrix substratum (Lauffenburger and Horwitz, 1996; Yamada and Sixt, 2019). Adhesion to the substrate is achieved through integrin binding with coordinated mechanosensory integration with intracellular focal adhesions. These substrate interactions potentiate, in part, the capacity of cells to sense differences in the material properties of the extracellular matrix (Yamada and Sixt, 2019). Cells integrate this adhesion information from the substrate with the mechanics of forward actin protrusion and actomyosin contractions to control cell polarity, migration directionality, and coordinated cell movements (Haeger et al., 2015).

Therefore, the direction of cell migration and commitment to a migratory phenotype necessitates a multivalent response to chemotactic and extracellular matrix-mediated stimuli. Growth factors affecting cell migration include platelet-derived growth factor (PDGF), fibroblast growth factor (FGF), insulin-like growth factor (IGF), and transforming growth factor (TGF)-β. PDGF, FGF, and IGF are present in post-traumatic cartilage (Mishima and Lotz, 2008; Hopper et al., 2015). Of these growth factors, PDGF is one of the most potent. In chondrocytes and mesenchymal stem cells, PDGF-BB and -AB induce a robust migratory response from the cells, with a lower response being elicited from PDGF-AA (Mishima and Lotz, 2008). PDGF is of special interest in biomedical studies as a prominent component of the serum used in cell culture media. TGF-β is also involved in the injury response of the cell, regulating the fibrogenic signaling axis. This TGF-β-mediated injury response is associated with the accumulation of cell surface and extracellular matrix proteoglycans (Cattaruzza and Perris, 2005).

The accumulation of membrane-associated proteoglycans after injury can have multiple roles including regulating growth factor-mediated signaling, ectodomain shedding, the synthesis of scaffolding proteins, the composition of the subcellular microdomain, and the localization of glycosaminoglycan chains to the cell. These modifications are associated with both anti-adhesion and anti-migratory effects and impact the regenerative and reparative potential of the tissue (Krekoski et al., 2001; Bradbury et al., 2002; Zuo et al., 2002). Specifically, sulfated GAG side chains exert anti-adhesion and anti-migratory effects on the cell due to the negative charge of the molecule (Davies et al., 2008). The anti-migratory effects of chondroitin sulfate chains in the cartilage is well-documented, particularly in the major proteoglycan of articular cartilage aggrecan (Hunziker and Rosenberg, 1996; Perris et al., 1996; Johnson et al., 2005; Johnson et al., 2006; Davies et al., 2008). Less is known about the role of other chondroitin sulfates containing membrane-associated proteoglycans in the migration of cartilage cells.

One of these proteoglycans is neuron glial antigen 2 (NG2; human homolog, CSPG4; mouse homolog, AN2). NG2/CSPG4 is a single-pass transmembrane proteoglycan with chondroitin sulfate chains present on the ectodomain. NG2/CSPG4 is present in cartilage (Fukushi et al., 2003; Yotsuya et al., 2019) and regulates the homeostasis of the cartilage during health and disease (Midwood and Salter, 1998; Midwood and Salter, 2001; Reed et al., 2022). NG2/CSPG4 has been implicated in cell migration and motility in other cell types (Fukushi et al., 2004; Makagiansar et al., 2007) but has not been studied in temporomandibular joint cartilage. NG2-/CSPG4-mediated migration/motility is achieved through both direct and indirect mechanisms. NG2/CSPG4 directly engages with the cell surface microdomain through beta-1 integrin (Burg et al., 1996; Burg et al., 1997; Goretzki et al., 1999; Fukushi et al., 2004; Makagiansar et al., 2007; Chekenya et al., 2008; Jamil et al., 2016) and is a cell surface receptor for type VI collagen (Stallcup et al., 1990; Nishiyama and Stallcup, 1993; Burg et al., 1996; Burg et al., 1997; Tillet et al., 2002; Huang et al., 2010). NG2/CSPG4 also regulates cell motility through binding with pro-migratory growth factors, PDGF-AA and FGF2 (Nishiyama et al., 1996a; Nishiyama et al., 1996b; Goretzki et al., 1999; Grako et al., 1999). Indirectly, the addition of NG2/CSPG4 enhances cellular motility even in cells that do not contain substantial endogenous cell surface NG2/CSPG4 such as vascular endothelial cells, promoting *in vitro* endothelial tube formation and *in vivo* blood vessel development (Fukushi et al., 2004).

The role of NG2/CSPG4 in the migratory potential of a cell is regulated by differential phosphorylation at two sites. Phosphorylation at Thr2256 through PKCα promotes migration by enhancing NG2/CSPG4 colocalization with beta-1 integrin on the leading edge lamellipodia. Phosphorylation at Thr2314 through ERK-1/2 enhances NG2/CSPG4 colocalization with β-1 integrin on the apical surface microprotrusions and promotes a proliferative phenotype (Makagiansar et al., 2007). The ERK-1/2 and PCKα signaling pathways are important mediators of chondrocyte differentiation and mechanotransduction (Zhen et al., 2001; Lee et al., 2002). PKCα activation occurs in an mTOR-dependent manner, with mTOR signaling being a potent upstream regulator of migration (Zhou and Wong, 2006). Chondrocyte migration is associated with the ERK-1/2 pathway. Together, the ERK, PKC, and mTOR pathways represent parallel and complementary signaling pathways regulating cell migration in cartilage (Fujita et al., 2004; Davies et al., 2008; Lu et al., 2013).

NG2/CSPG4 contains large chondroitin sulfate chains, suggesting that it should have anti-migratory effects on the cells. However, multiple studies have demonstrated that the presence of NG2/CSPG4 in cancers is a strong indicator of the metastatic potential of the tumor (Burg et al., 1997; Burg et al., 1998; Chekenya and Pilkington, 2002), with NG2/CSPG4 loss of function experiments resulting in attenuated cell migration (Cattaruzza et al., 2013b; Jamil et al., 2016; Yang et al., 2019a; Wilms et al., 2022). Given the multivalent nature of NG2-/CSPG4-dependent migratory signaling, with cytoskeletal-mediated/growth factor-independent pathways and convergent growth factor/extracellular matrix pathways potentially influencing the phenotype and behavior of the cell (Cattaruzza and Perris, 2005), the role of NG2/CSPG4 as a regulator of cell migration in cartilage health and disease has yet to be resolved.

The interaction of NG2/CSPG with OMI/HTRA2 to regulate oxidative stress implicates the proteoglycan in the transcriptional regulation of the cell stress response (Maus et al., 2015). Oxidative stress is an important factor in the progression of degenerative conditions such as osteoarthritis (Tanaka et al., 2008). Oxidative stress can be modeled in an *in vitro* environment using serum starvation (Tangtrongsup and Kisiday, 2018), not only elevating reactive oxygen species but also removing a number of key growth factors known to interact with NG2/CSPG4, synchronizing the cell-cycle kinetics of cells, suppressing proliferation, and altering signaling molecules associated with NG2/CSPG4 functionality such as ERK-1/2, PKCα, and mTOR signaling (Hasan et al., 1999; Chen and Long, 2014; Raja et al., 2018). Here, we will leverage this cell culture model together with live cell imaging of migration to determine the role of NG2/CSPG4 as a regulator of cell migration during normal physiological and cell stress conditions.

## 2 Materials and methods

### 2.1 Control, NG2/CSPG4 knockout, and DsRed NG2/CSPG4 reporter mice

Control mice from a C57BL/6J background were purchased from Jackson Laboratory. Knockout mice were acquired from the KOMP repository (Cspg4tm1a(KOMP)Wtsi/Bcm) and were generated using the knockout-first allele, promoter-driven section kit. The knockout mice used in this study were generated by cross-breeding with a Cre-expressing line to generate a reporter-tagged deletion allele. Heterogeneous mice with the reporter-tagged deletion allele were backcrossed with a C57BL/6J line and then mated to generate a reporter-tagged deletion allele for NG2/CSPG4. All animals were housed together to minimize confounding conditions. NG2/CSPG4 knockout mice were viable through skeletal maturity with no strong developmental phenotypes aside from those reported, including increased lean body mass (Mousephenotype.org). There is a mild but significant phenotype in the TMJ cartilage (Reed et al., 2022). DSRed-NG2/CSPG4 (DSRed|NG2) reporter mice were acquired from a commercial source (Tg(Cspg4–DsRed.T1)1Akik, Jackson Laboratory) and bred to generate offspring hemizygous for the reporter construct. TMJ osteoarthritis was induced using the unilateral partial discectomy model as previously described (Yotsuya et al., 2019; Reed et al., 2022). The use of all animal tissues followed an approved animal use protocol (UIC ACC #23-042/#20-068).

### 2.2 Immunohistochemistry and immunocytochemistry

For immunohistochemistry, the sections were deparaffinized, treated with sodium borohydride (132.2 mM, 452882, Sigma), permeabilized with methanol and 0.5% Triton (v/v), blocked in 5% donkey serum (D9663, Sigma, St. Louis, MO) for 2 h, and incubated with primary antibodies against NG2/CSPG4 (1:200, AB5320, Sigma Millipore, Santa Cruz, CA) or a custom monoclonal antibody raised against the NG2/CSPG4 intracellular domain (1:200, GGQPDPELLQFCRTPNPALRNGQYWV, UIC Protein Core, Chicago, IL, United States). Secondary labeling was done with Alexa Fluor donkey anti-rabbit 568 (1:500, Invitrogen, Carlsbad, CA). Nuclei were labeled with DAPI (D9542-1 MG, 1 μg/μL, Sigma, St. Louis, MO). The sections were imaged using an inverted fluorescence microscope using a ×10 objective (DMI6000B, Leica, Buffalo Grove, IL). Laser intensity, gain, and magnification were standardized for all acquisitions. Brightness and contrast settings were standardized for all images during post-processing. All data were compared to those of a no primary antibody control and isotype control. For immunocytochemistry, the cells were fixed using HistoChoice tissue fixative (VWRVH102, VWR, Radnor, PA). Antigen retrieval is the same as that described for immunohistochemistry. All samples were imaged using a laser scanning confocal microscope with a ×63 oil immersion objective (LSM 710, Zeiss) using identical laser intensity, brightness, and gain standardized for all image acquisitions. The images are representative of four biological replicates for each experimental group.

### 2.3 Primary cell isolation

Primary cell isolation was carried out by following the published methods for chondrocytes (Gosset et al., 2008; Reed et al., 2021; Reed et al., 2022; Bagheri Varzaneh et al., 2023). Mandibular condylar cartilages (MCCs) were collected from wild-type, NG2/CSPG2 knockout, and/or DsRed reporter mice from 10–14-day-old pups. Extracted MCCs were placed in collection medium DMEM (12492–013, Gibco, Gaithersburg, MD) and rinsed twice with sterile phosphate-buffered saline (PBS) solution with 25 mg/mL Plasmocin (ant-mmp, InvivoGen, San Diego, CA), 50 U/mL penicillin, and 0.05 mg/mL streptomycin (P0781, Sigma, St. Louis, MO) under a sterile flow hood. For cell isolation, MCCs were digested in type II collagenase (S004174, Worthington Biochemical, Lakewood, NJ) suspended in 3 mg/mL Dulbecco’s modified Eagle medium (11966–025, Gibco, Gaithersburg, MD) for 45 min in a thermal incubator under 5% CO_2_ at 37°C. The tissue fragments were agitated using a pipette to detach soft tissues and were then washed with PBS solution. The cartilage pieces were retrieved and then transferred to 1.5 mg/mL type II collagenase digestion medium overnight in 5% CO_2_ at 37°C. The cell solution was retrieved and placed in a 15-mL tube and then dispersed by using a transfer pipette. The cell suspension was filtered using a sterile 48 uM cell strainer and then centrifuged at 10,000 g for 10 min at room temperature. The pellet was retrieved and washed with PBS and resuspended with 15 mL of the culture medium. The cell density was calculated using a hemocytometer. All cells were cultured, grown to confluence, and used before the third passage.

### 2.4 Bulk RNA-seq with gene ontology analysis

The bulk RNA-seq analysis follows previously published methods (Reed et al., 2022). In short, RNA was isolated using the Qiagen RNeasy Mini Kit (79216 Qiagen, Germantown, MD). A Poly(A) RNA sequencing library was prepared with Illumina’s TruSeq Stranded mRNA sample preparation protocol, including oligo-(dT) magnetic bead purification, poly(A) RNA fragmentation, DNA library construction, and Agilent Technologies 2100 Bioanalyzer high-sensitivity DNA chip quality control. Sequencing was performed using Illumina’s NovaSeq 6000 sequencing system. HISAT2 was used to carry out mapping of reads for the genome, and the reads were assembled using StringTie. All transcriptomes were merged using perl scripts and gffcompare. StringTie and edgeR were used to estimate the expression levels and perform mRNA expression levels. Three biological replicates were used for the analysis. Differential gene expression was used for a Gene Ontology analysis using the ShinyGO platform (Ge et al., 2020) using the biological processes pathway database (ShinyGO 0.77), with differentially up- and downregulated genes analyzed together (*q*-value <0.05) against background genes.

### 2.5 Western blot

For *in vitro* and *in vivo* protein isolation of cultured cells, the plates were washed in ice-cold, 1× PBS, lysed using an extraction reagent (M-PER, 78501, Thermo Fisher, Waltham, MA) with protease (cOmplete, 4693116001, Sigma, St. Louis, MO) and phosphatase (PhosSTOP, 4906845001, Sigma, St. Louis, MO) inhibitors. For *in vitro* protein isolation of the cell–agarose scaffolds, the samples were rinsed in 1× PBS for 20 min, placed in Laemmli buffer, boiled for 5 min, cooled on ice, and spun down for 2 h using a mini-spin column (Pierce Spin Cups, 69700, Thermo Fisher, Waltham, MA). For all samples, insoluble lysates were removed by centrifugation at 14,000 *g* for 15 min at 4°C. For the monolayer cell and tissue samples tested for NG2/CSPG4, the supernatant was incubated with chondroitinase ABC (100330-1, AMSBio, Cambridge, MA) added at 0.05 units/mL for 3 h at 37°C. For all samples, the lysates were adjusted to a 4x Protein Sample Loading Buffer (928–40004, LI-COR, Lincoln, NE), heated at 100°C for 5 min, run on a 4%–15% sodium dodecyl sulfate polyacrylamide gel (SDS–PAGE), and analyzed using Western blot with antibodies against NG2/CSPG4 (1:500, AB5320, Sigma Millipore, Santa Cruz, CA) and PCNA (1:1000, 2586S, Cell Signaling Technologies, Danvers, Massachusetts). The blots were imaged using a LI-COR fluorescence quantitative western blot. Fluorescence values were normalized to β-actin and standardized to experimental control samples. Four biological replicates were used for all western blot.

### 2.6 Migration assay

Primary cells under three passages were used for the study. Primary cells were trypsinized and seeded in a three-well culture insert for cell migration (ibidi, Gräfelfing) at a concentration of 55 × 103 cells per 100 ul. The cells were incubated in secondary growth conditions overnight to achieve an 80% confluence. To suppress cell proliferation, mitomycin C (5 μg/mL; Sigma-Aldrich, St. Louis, MO) was added to cell culture media, and the cells were incubated for 2 h at 37°C at 5% CO_2_. To initiate cell migration, the insert was removed and the culture plate was placed in a live-cell microscope stage at 37°C and 5% CO_2_ (Leica, DMI6000B). The cells were imaged under phase contrast microscopy every 15 min for 24 h. Each experimental group consisted of four biological replicates and four technical replicates per sample. To quantify cell migration, the gap closure rate was analyzed using ImageJ software. The leading edges of the cells on each image were manually traced to create an outline of the target cell-free areas at 0, 4, 12, and 24 h. The ImageJ measure tool was used to calculate the areas in pixels at each time point and were transferred to the data window. The cell-free area at 0 h is used as a baseline to calculate % closure of the area over time: % closure = [(cell-free area at 0 h) − (cell-free area at x h)]/(cell-free area at 0 h). Cell counting was performed by counting the total number of cells in the cell-free zone defined at time 0 for each experimental time point. Four biological replicates and two or four technical replicates were used for each experimental group.

### 2.7 RT-qPCR

For quantifying gene expression changes, RNA was isolated using the RNeasy Mini Kit (74104, Qiagen, Germantown, MD). All target genes were amplified with the SYBR^®^ Select Master Mix (4385610, Applied Biosystems, Waltham, MA) in a Bio-Rad iQ5 (Bio-Rad, Des Plaines, IL). Primer sequences are reported in Supplementary Table S3. Validation of all primers using negative controls substituting molecular grade water for cDNA was carried out for each primer for standard quality control. Gene expression changes were calculated by the comparative threshold cycle method with data standardized to a sample control and normalized to GAPDH using the ΔΔCq method. Negative controls with no cDNA were run for all primers. Four biological replicates and two technical replicates were used for each experimental group.

### 2.8 Cell tracking

Primary cells from the mandibular condyles of DSRed|NG2 reporter mice were plated in a 12-well plate and grown to confluence. For the migration assay, a scratch was made across the length of the plate with the tip of a 10-μL pipette, creating a higher signal intensity of DsRed positive cells at the injury site than using the insert. The cells were cultured in serum-supplemented and low-serum condition during the migration assay. Live-cell microscopy followed the methods previously described with a fluorescent channel (588 excitation/583 emission). Laser intensity, brightness, and the contrast of image acquisition were established before starting the experiment to optimize imaging DsRed-positive cells. Exported data were processed and cell motility performance was quantified using TrackMate (Tinevez et al., 2017). DsRed-positive and -negative cells were tracked using the software and manually correct by the frame. The migration, velocity, and distance of each cell were calculated and output by TrackMate. Four biological replicates and five technical replicates (i.e., cells) were used for each experimental group.

### 2.9 Immortalization of mandibular fibrochondrocytes

Primary cells from a 10–14-day-old mouse mandibular condylar cartilage were isolated as described in the previous section. Once confluent in a 100-mm dish, the cells were transduced with the supernatant of a recombinant retroviral vector containing cDNAs expressing hTERT containing a GFP-expressing protein. After one round of retroviral transduction, the cells were given serum-supplemented media and grown to confluence. Confluent cells were then trypsinized to a single-cell suspension and sorted for GFP using a flow cytometer (Bio-Plex, Bio-Rad). GFP-positive cells were then plated in a 96-well plate using a single-cell cloning approach. The clone with the highest growth rate was selected and frozen for preparing a stock solution. The resuspended cells from this clone were used for all experiments and for the CRISPR/Cas9 modifications from cells under 10 passages.

### 2.10 CRISPR/Cas9 modification of the NG2/CSPG4 ectodomain

The collagen VI binding sequence for NG2/CSPG4 was identified from published sequences (Tamburini et al., 2018). The sgRNA sequences were designed from this target sequence using the ALT-R^®^ CRISPR–Cas9 system from Integrated DNA Technologies (IDT, Coralville, IA, United States). All of the off- and on-targets were designed using IDT software (https://eu.idtdna.com/pages). The ALT-R^®^ CRISPR–Cas9 system was used for transfection and includes the Cas9 protein, trans-activating CRISPR RNA (tracrRNA), and CRISPR RNA (crRNA). TracrRNA (5 nmol; 3 μL) was mixed with target-specific crRNA (2 nmol, 3 μL) in the IDT nuclease-free duplex buffer. The solution was incubated for 5 min at 95°C and slowly annealed at room temperature for 10 min. The sgRNA (400 ng in total) and Cas9 (1 μg) were complexed for 5 min in reduced serum medium (Opti-MEM, Gibco, NY, United States). The solution was combined with 150,000 cells, placed in a cuvette, and electroporated at 225 V for six pulses (Gene Pulser Xcell, Bio-Rad) following the published optimization values for chondrocytes (Schönenberger et al., 2011). The cells were then incubated in 10% FBS/DMEM for a further 48 h and allowed to recover. After the recovery period, dead cells were removed and adherent cells were trypsinized to generate a single-cell suspension. These cells were then plated for single-cell clonal expansion using the dilution method. Clone colonies were expanded into two plates. One plate was screened by PCR with primers designed to span the target deletion (Table 1). The second plate was screened using Sanger sequencing (3730xl Analyzer, Life Technologies). NG2/CSPG4 sequences from the control and CRISPR/Cas9 truncated cells were manually aligned to confirm the truncation of the amino acid sequence corresponding to the collage VI binding region. One clone with the best growth properties and the appropriate genotype was isolated and used for all subsequent studies.

### 2.11 Statistical analysis

A one-way ANOVA was used for all statistical tests. *Post hoc* Bonferroni tests were carried out for multi-group comparisons (SPSS, Chicago, IL). A *p*-value of <0.05 was considered statistically significant for all studies.

## 3 Results

**FIGURE 1:**
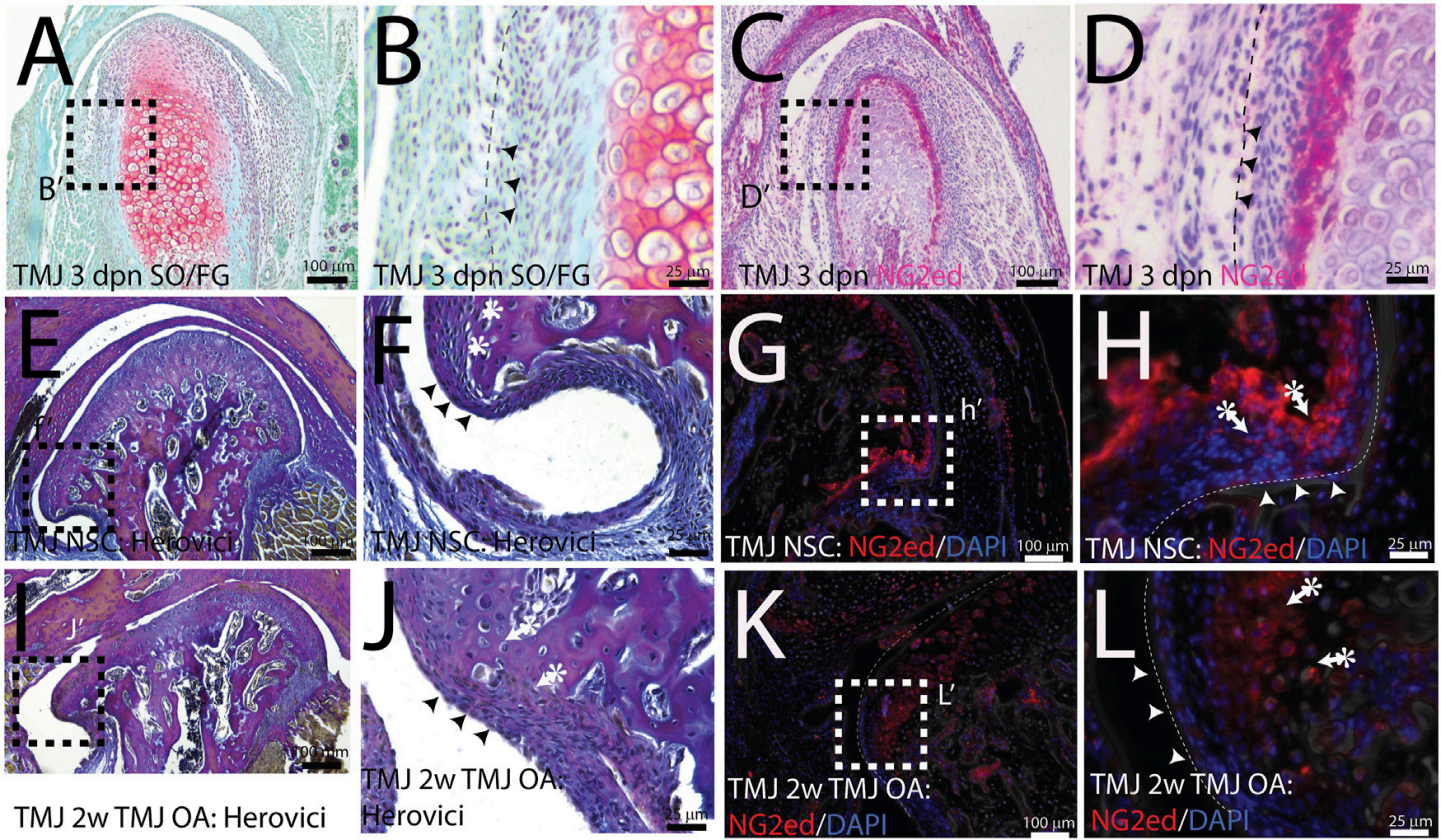
NG2/CSPG4 is abundant in a restricted population of cells in the mandibular condyle. **(A, B)** Safranin O/Fast Green staining of a 3-day post-natal mouse TMJ illustrating that the superficial fibrous layer of the condylar cartilage (arrows) and an underlying prechondroblastic layer. The chondroblastic layer is delineated by the presence of proteoglycans (red). **(C, D)** Immunohistochemistry illustrating that NG2/CSPG4 is strongly expressed in the prechondroblastic layer. **(E, F)** Herovici’s polychrome stain of the pole of the mandibular condyle illustrating that the fibrous layer of the bony periosteum is continuous with the articular layer of the mandibular cartilage and that the underlying prechondroblastic layer is continuous with the osteogenic layer of the bony periosteum; **(G, H)** immunofluorescent staining illustrating that NG2/CSPG4 is localized to the prechondroblastic cells in the mandibular condylar cartilage and on a cell layer adjacent to the bone in the osteogenic layer of the condylar periosteum. **(I, J)** Herovici’s polychrome stain of early-stage TMJ OA illustrating an increase in the thickness of prechondroblastic/chondroblastic layers at the pole of the condyle and a thickening of the periosteum. **(K, L)** Immunofluorescence staining of NG2/CSPG4 in early-stage TMJ OA illustrating an increase in immune-positive cells in the prechondroblastic layer of the mandibular condylar cartilage and negligible staining in the fibrous and osteogenic layers of the periosteum.

NG2/CSPG4 is abundant in a restricted cell population in the mandibular condylar cartilage. The mandibular condylar cartilage of the TMJ is a secondary cartilage that forms from a migratory cell population derived from the periosteum of the developing mandibular mesenchyme (Hinton and Carlson, 2005). In the mandibular condylar cartilage, the superficial perichondrium is continuous with the fibrous bony periosteum and the underlying prechondroblastic layer is continuous with the osteogenic layer of the bony periosteum. Both of these cell layers are superficial to chondroblastic cells evident by the presence of proteoglycans such as aggrecan (Figures 1A, B). During MCC development, NG2/CSPG4 is abundant in the prechondroblastic layer (Figures 1C, D). In the skeletally mature mandibular condylar cartilage, the continuity of the perichondrium with the periosteum is still apparent near the pole of the condyle (Figures 1E, F). There is also an NG2/CSPG4-positive cell population deep in this perichondrium, in the prechondroblastic and chondroblastic cell layer (Figures 1G, H). During the early stages of TMJ OA, the pole of the condyle expands as the condyle flattens and the perichondrium thickens (Figures 1I, J), with a concomitant increase in the NG2/CSPG4-positive layer deep in the perichondrium (Figure 1K, L). These data illustrate that NG2/CSPG4 is not localized to the fibrous perichondrium but is concentrated in cells that have committed to chondrogenic differentiation.

**FIGURE 2:**
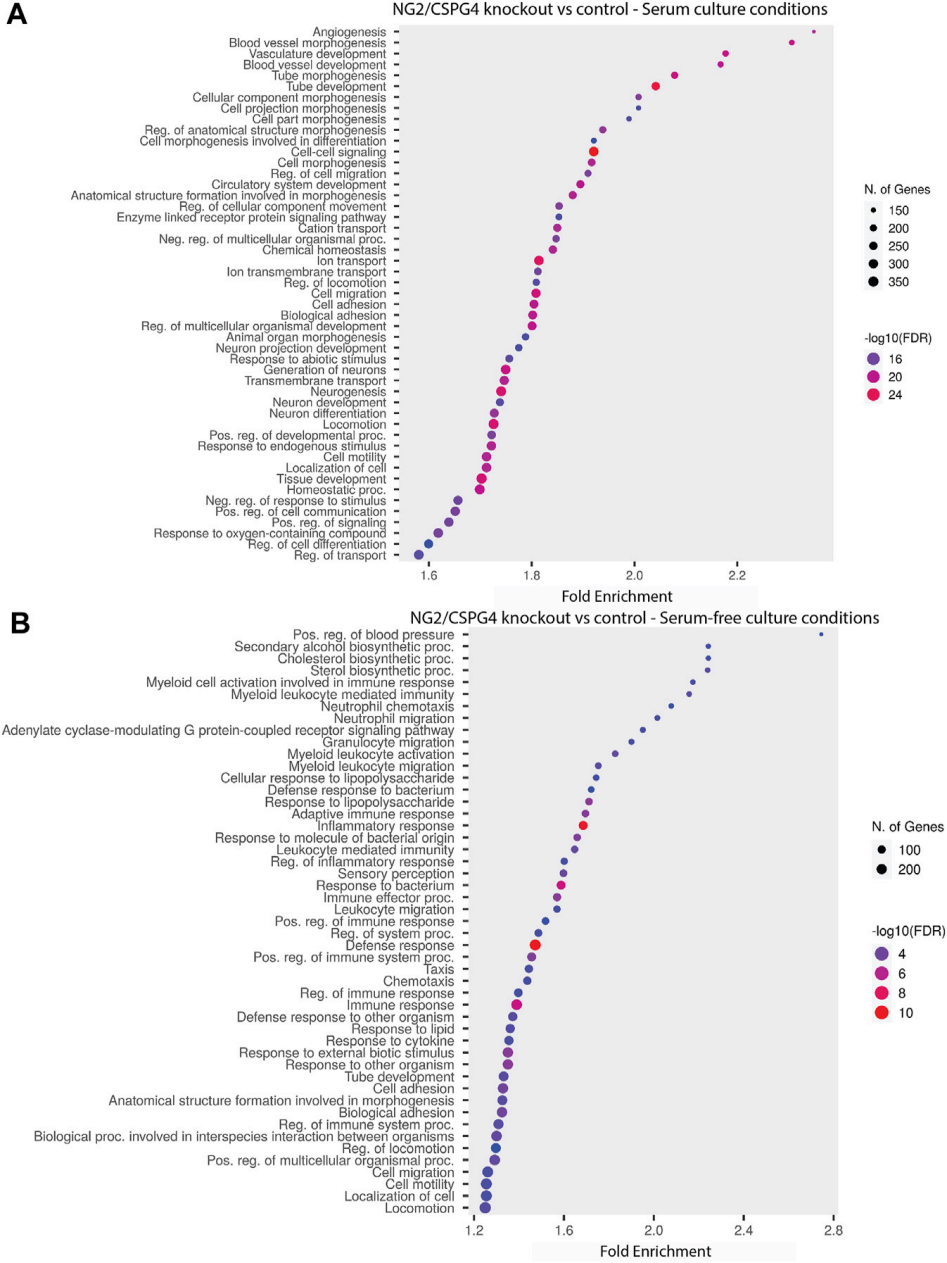
NG2/CSPG4 regulates the transcriptional control of cell-matrix interactions and migration in serum-supplemented and serum starvation conditions. **(A)** Gene Ontology enrichment analysis comparing the differences in the biological process terms from a comparison of NG2/CSPG4 knockout and control c57 primary mandibular condylar cartilage cells in serum **(A)** and serum-free **(B)** cell-culture conditions. Data represent sequencing results from *n* = 3/genotype per treatment group.

NG2/CSPG4 regulates the transcriptional control of cell-matrix interactions and migration. The bulk RNA-seq analysis of primary mandibular fibrochondrocytes derived from the mandibular condylar cartilage of the control and NG2/CSPG4 knockout mice cultured in serum-supplemented and serum starvation conditions was analyzed using Gene Ontology (GO) enrichment analysis. Using the biological process ontology set, several significant GO terms associated with cell migration during serum culture conditions were identified, including the “Regulation of cell migration” (-log_10_ FDR = 16; 200 genes), “Cell migration” (–log_10_ FDR = 24; 300 genes), and “Cell motility” (–log_10_ FDR = 16; 300 genes). In serum starvation conditions, significant pathways include “Cell migration” (–log_10_ FDR = 4; 200 genes) and “Cell motility” (–log_10_ FDR = 4; 200 genes). In both serum-supplemented and serum starvation conditions, NG2/CSPG4 knockout cells have a transcriptionally distinct profile affecting genes that regulate cell migration (Figure 2).

**FIGURE 3:**
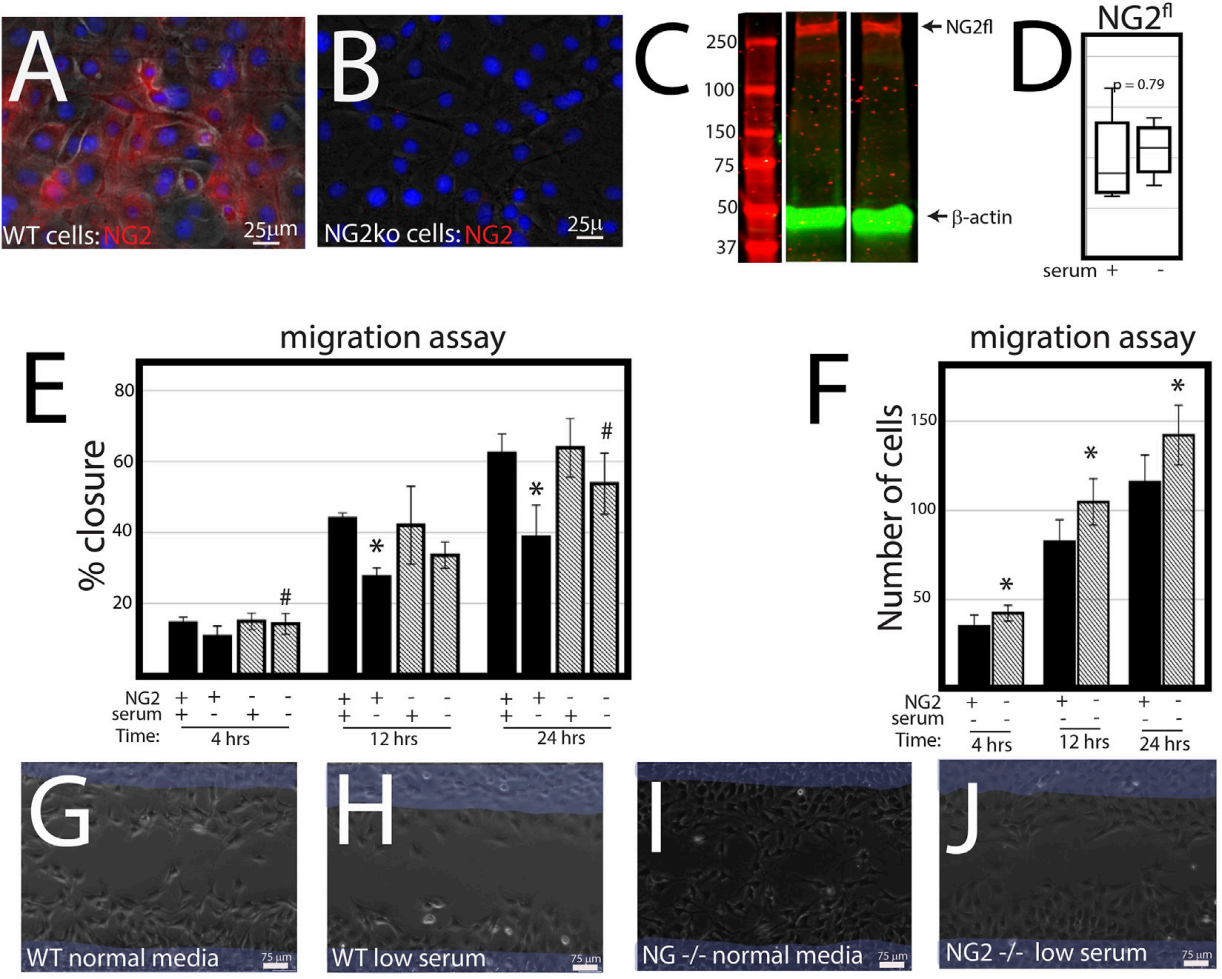
NG2/CSPG4 knockout cells migrate more quickly in serum starvation conditions. **(A)** Immunocytochemistry with immunostaining for NG2/CSPG4 in control primary mandibular fibrochondrocytes. **(B)** Immunocytochemistry with immunostaining for NG2/CSPG4 in NG2/CSPG4 knockout primary mandibular fibrochondrocytes. **(C)** Western blot of NG2/CSPG4 from the protein collected from control primary mandibular fibrochondrocytes cultured in serum-supplemented and serum starvation conditions. The full-length NG2/CSPG4 fragment is indicated at 300 kDa. Continuous western blot are provided in Supplementary Figure S1. **(D)** Quantification of the full length NG2/CSPG4 fragment with values standardized to β-actin and fold-change is calculated relative to the serum supplemented condition. *n* = 4/experimental group. **(E)** Quantification of a migration assay from the control and NG2/CSPG4 knockout primary mandibular fibrochondrocytes in serum-supplemented and serum starvation conditions measured at 4, 12, and 24 h. Data represent the percent closures of the cell-free region relative to time 0. **(F)** Quantification of the number of cells that passed in the cell free from the migration assay from serum starvation conditions at 4, 12, and 24 h. **(G–J)** Raw data visualization of the migration data with the cell-free range at time 0 indicated in yellow, superimposed over the same region after 24 h of migration. For all graphs, * represents the comparisons of serum-supplemented and serum starvation conditions with * = *p* < 0.05; # represents comparisons of the control versus NG2/CSPG4 knockout cells with # = *p* < 0.05.* = *p* <0.05 and *** = *p* <0.001; # represents comparisons of serum-supplemented and serum starvation conditions with # = *p* < 0.05 and *** = *p* < 0.001; # represents the comparisons of the control versus NG2/CSPG4 knockout cells with # = *p* < 0.05 and ### = *p* < 0.001. Representative live-cell videos of migration are provided as Supplementary Videos S1–S4.

NG2/CSPG4 knockout cells migrate more quickly under serum starvation conditions. Control and NG2/CSPG4 knockout cells cultured in serum-supplemented media and in serum starvation conditions illustrate that NG2/CSPG4 is only present on the control cells (Figures 3A, B). Western blot analysis of the control cells illustrates that serum starvation does not affect the amount of full-length NG2/CSPG4 protein (Figures 3C, D). In serum-supplemented cell culture conditions, there was no difference in cell migration between the control and NG2/CSPG4 knockout cells measured by the percent closure or cell-free area after 24 h of migration (Figure 3E). In serum starvation conditions, NG2/CSPG4 knockout cells migrated more than the controls, as measured by both the percent closure (Figure 3E; *p* <0.05; *n* = 4/genotype) and the number of cells in the cell-free region after 24 h (Figure 3F; *p* <0.05; *n* = 4/genotype). Furthermore, there was no significant difference in the percent closure in NG2/CSPG4 knockout cells in serum-supplemented and serum-free culture conditions (Figure 3E).

**FIGURE 4:**
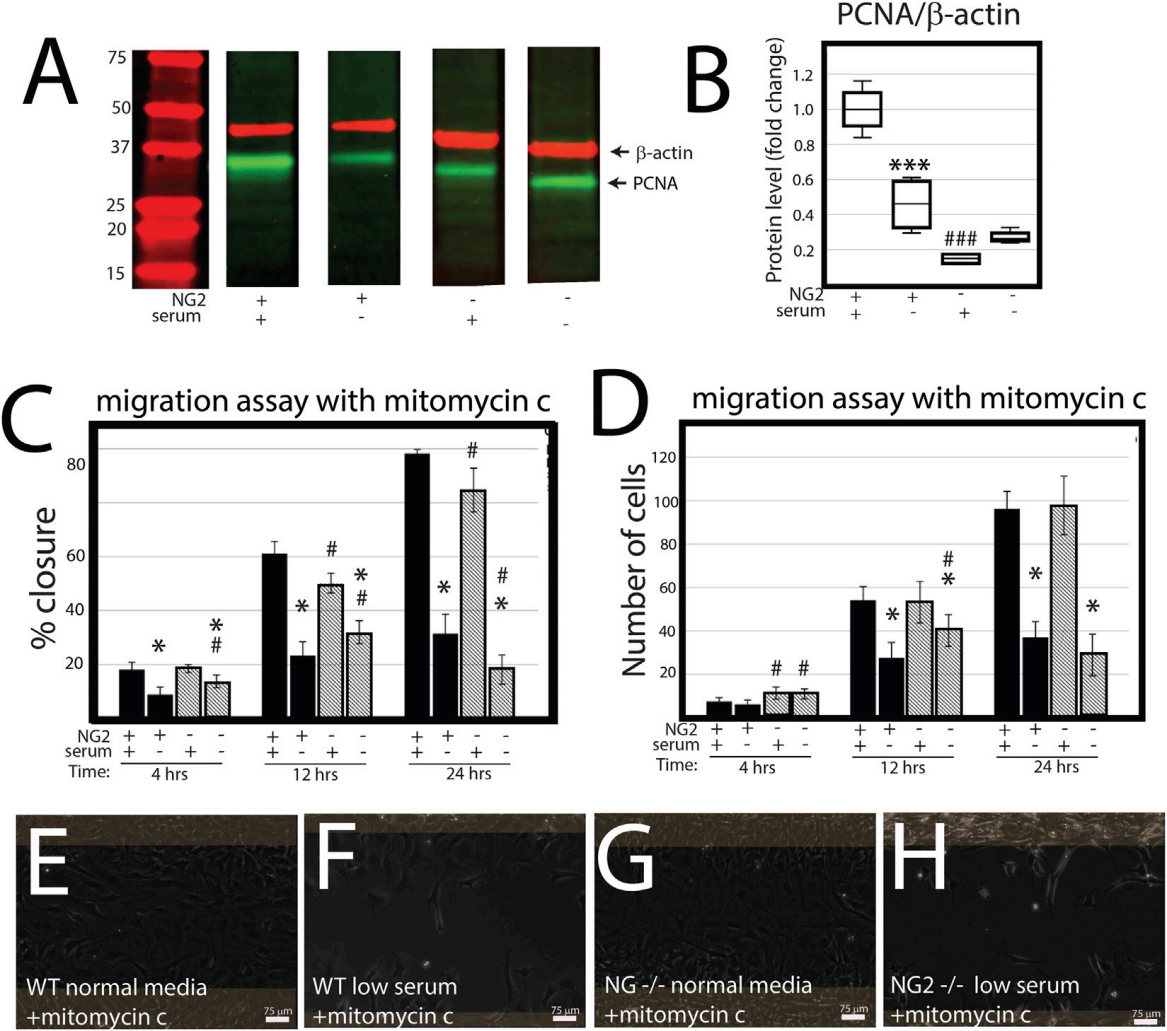
NG2/CSPG4 knockout cells have suppressed proliferation, but the rate of migration occurs in a proliferation-independent manner. **(A)** Western blot of PCNA from protein collected from the control and NG2/CSPG4 knockout primary mandibular fibrochondrocytes cultured in serum-supplemented and serum starvation conditions. The bands are representative of *n* = 4/experimental group. Continuous Western blots are provided in Supplementary Figure S1. **(F)** Quantification of PCNA with values standardized to β-actin and fold change is calculated relative to the serum-supplemented serum condition. **(C)** Quantification of a migration assay from the control and NG2/CSPG4 knockout primary mandibular fibrochondrocytes in serum-supplemented and serum starvation conditions measured at 4, 12, and 24 h pretreated with 5 μg/mL mitomycin C to suppress proliferation. Data represent the percent closures of the cell-free region at time 0. **(D)** Quantification of the number of cells that passed in the cell free region from the migration assay from serum starvation conditions at 24 h. **(E–H)** Raw data visualization of the migration data with the cell-free range at time 0 indicated in yellow, superimposed over the same region after 24 h of migration. For all graphs, * represents comparisons of serum-supplemented and serum starvation conditions with * = *p* < 0.05 and *** = p < 0.001; # represents the comparisons of the control versus NG2/CSPG4 knockout cells with # = *p* < 0.05 and ### = *p* < 0.001.

NG2/CSPG4 knockout cells migrate more quickly in a cell proliferation-independent manner. NG2/CSPG4 cells have significantly lower levels of cell proliferation during serum-supplemented cell culture conditions (Figures 4A, B; *p* <0.001; *n* = 4/experimental group). Serum starvation suppresses cell proliferation in both the control and NG2/CSPG4 knockout cells, equilibrating proliferation to statistically indistinguishable, but non-zero levels. To remove the confounding impact of proliferation, we repeated all migration experiments following 2 h of 5 μg/mL mitomycin C treatment to suppress DNA synthesis. In serum-supplemented cell culture conditions, mitomycin C pretreatment resulted in a decrease in the rate of cell migration in NG2/CSPG4 knockout cells when compared with the control as measured by the percent closure or cell-free area after 24 h of migration (Figure 4C; *p* < 0.05; *n* = 4/experimental group). In serum starvation conditions, mitomycin C pretreatment results in an increase in the rate of cell migration in NG2/CSPG4 knockout cells when compared with controls, as measured by both the percent closure (Figure 4C; *p* <0.05; *n* = 4/genotype) and an increase in the number of cells in the cell-free region after 4 and 12 h (Figure 4D; *p* <0.05; *n* = 4/genotype). The percent closure and number of cells in the NG2/CSPG4 knockout cells decreased at 24 h, likely due to elevated levels of cell death resulting from prolonged exposure to the stress conditions resulting from mitomycin C pretreatment and serum starvation (see Figure 4F).

**FIGURE 5:**
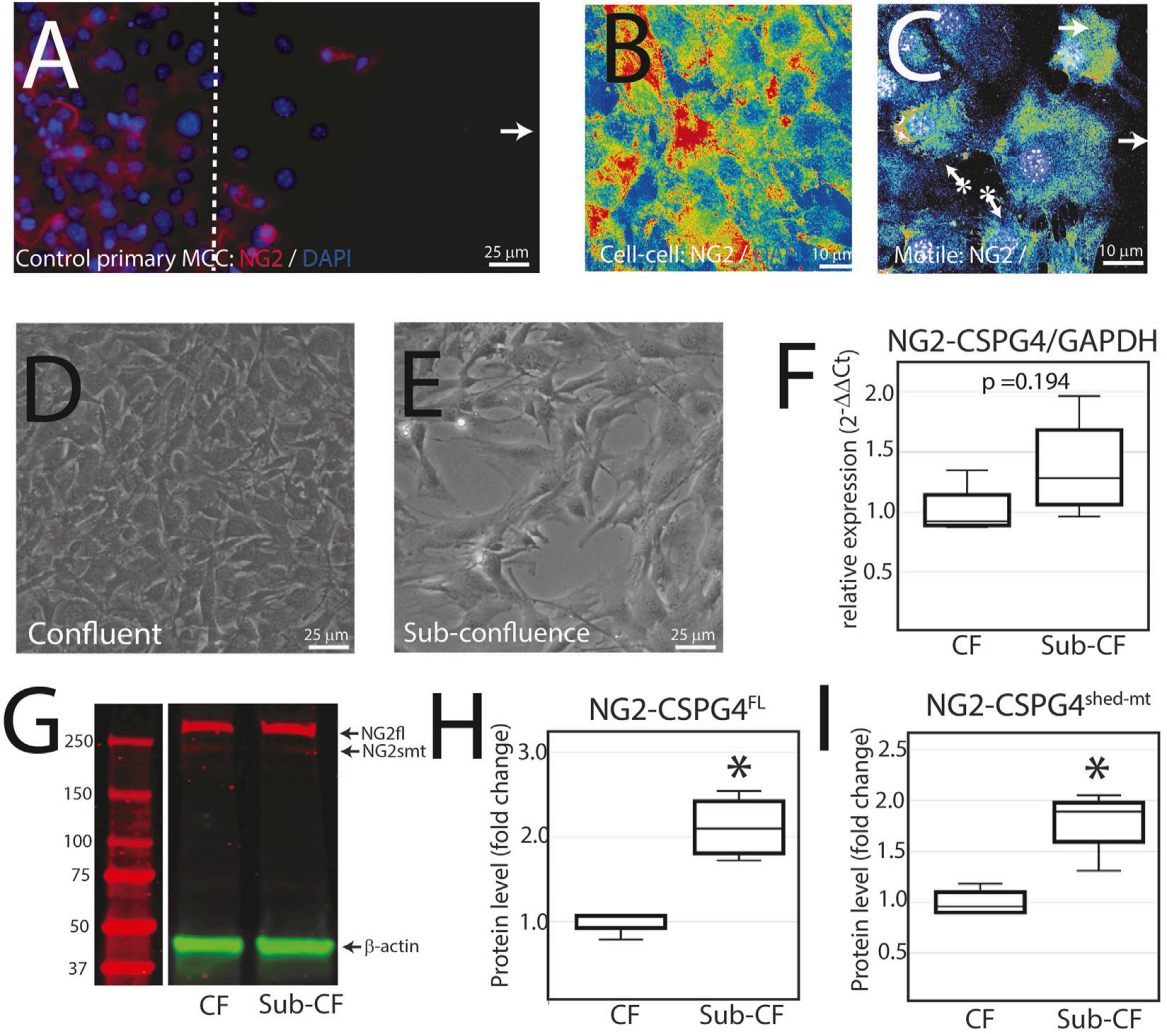
NG2/CSPG4 is diminished during active migration in mandibular fibrochondrocytes. **(A)** Immunocytochemistry of primary control mandibular fibrochondrocytes fixed and immuno-labeled with NG2/CSPG4 6 h after the start of migration. **(B)** Confocal immunofluorescence of NG2/CSPG4 pseudo-colored to illustrate the signal intensity from a region of the migration assay with high cell–cell contact. **(C)** Confocal immunofluorescence of NG2/CSPG4 pseudo-colored to illustrate the signal intensity from a region of the migration assay with actively migrating cells. **(D)** Bright-field microscopy of control primary mandibular fibrochondrocytes from a confluent plate. **(E)** Bright-field microscopy of control primary mandibular fibrochondrocytes from a sub-confluent plate. **(F)** RT-qPCR illustrating gene expression changes in NG2/CSPG4 in the confluent (CF) and sub-confluent (sub-CF) samples of control primary mandibular fibrochondrocytes. **(G)** Western blot of NG2/CSPG4 from the protein collected from confluent and sub-confluent samples of control primary mandibular fibrochondrocytes. The full-length fragment is indicated at 300 kDa and the shed membrane-tethered fragment is indicated at 275/260 kDa. These bands are representative of an *n* = 4/experimental group. Continuous blots are provided in Supplementary Figure S2. **(H–I)** Quantification of full-length NG2/CSPG4 with values standardized to β-actin and fold change is calculated relative to the confluent experimental group. * represents the comparisons of confluent and sub-confluent experimental groups with * = *p* <0.05.

NG2/CSPG4 is diminished during active migration in mandibular fibrochondrocytes. To characterize NG2/CSPG6 levels in actively migrating cells, we conducted a migration assay on a glass slide and fixed the cells after 6 h. Confocal immunofluorescence using an antibody against full-length NG2/CSPG4 illustrates that cell–cell contact is associated with high levels of NG2/CSPG4, while migration is associated with lower levels of NG2/CSPG4 (Figures 5A–C). It has been previously reported that NG2/CSPG4 levels are linked with cell density (Asher et al., 2005). To determine if the heterogeneous distribution of NG2/CSPG4 protein in these cells was related to cell density or the migratory phenotype, we characterized NG2/CSPG4 levels from a confluent and sub-confluent population of primary mandibular fibrochondrocytes (Figures 5D, E). There was no difference in the gene expression levels between the confluent and sub-confluent population (Figure 5F; *p* = 0.194; *n* = 4/experimental group). Western blot analysis illustrates that the sub-confluent population of cells had a significantly higher level of both full-length and shed-membrane tethered fragments of NG2/CSPG4 (Figures 5.G–I; *p* <0.05; *n* = 4/experimental group). Together, these findings illustrate that the turnover of the NG2/CSPG4 protein in active migration cells is a property of the migratory phenotype and not of the density of the cells.

**FIGURE 6:**
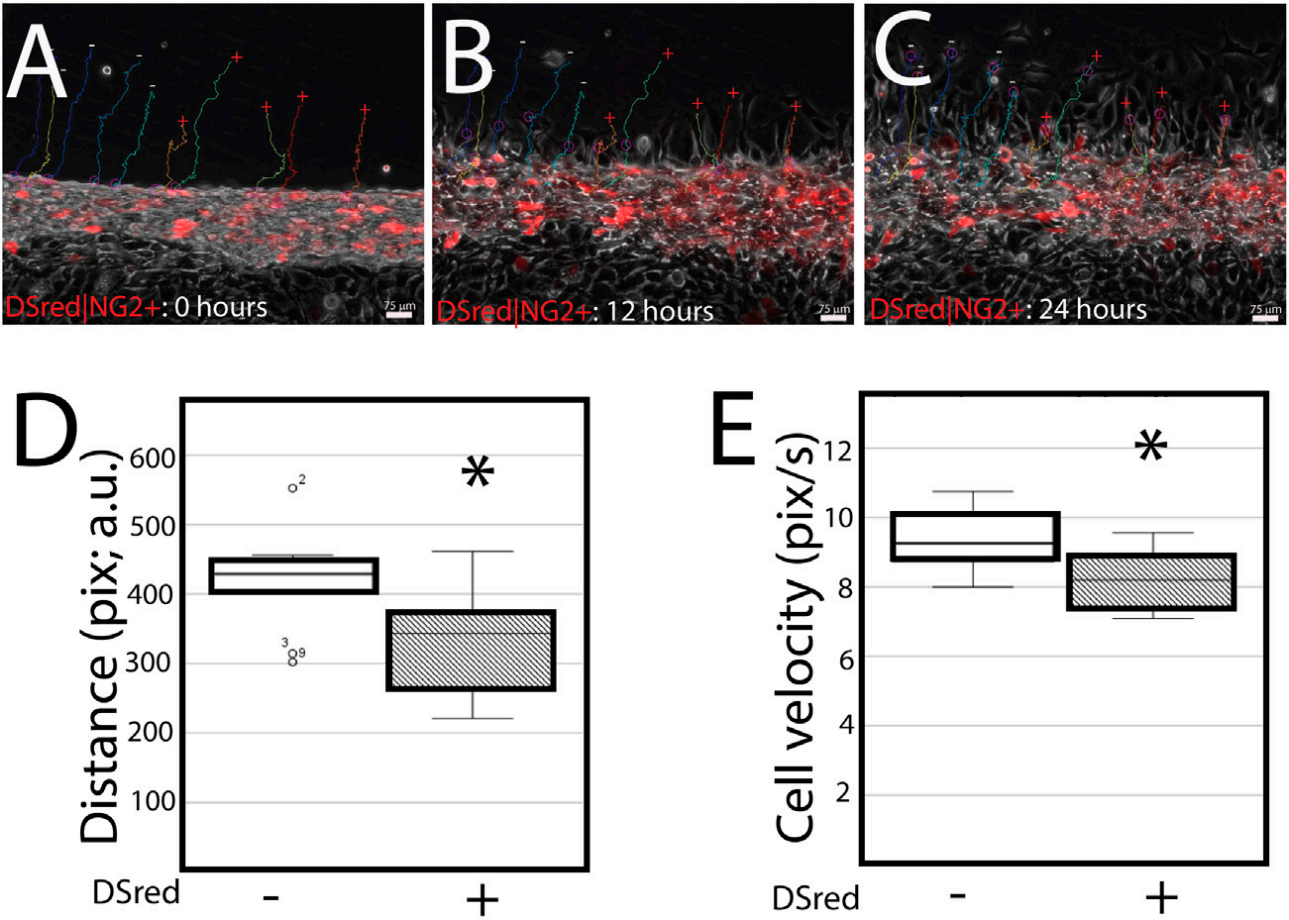
DSRed|NG2-positive cell migrate slower in serum starvation conditions. **(A–C)** Cell migration dynamics after a scratch assay at 0 **(A)**, 12 **(B)**, and 24 **(C)** hours. DSRed|NG2-positive are red. The migration path of DSRed|NG2-positive (+) and -negative (−) cells is superimposed over each image. **(D)** Quantification of the distance traveled by each DSRed|NG2-positive and -negative cell reported in pixels, arbitrary units. **(E)** Quantification of the velocity of each cell in DSRed|NG2-positive and -negative cells reported in pixels/second, arbitrary units. For all experiments, data were collected from two biological replicates, with five DSRed|NG2-positive and -negative cells quantified from each biological replicate. * represents the comparisons of DSRed|NG2-positive and -negative cells with *p* <0.05. A representative live-cell video of cell migration is provided as Supplementary Video S05.

DSRed|NG2-positive cells migrate slower in serum starvation conditions. Since NG2/CSPG4 knockout cells have a distinct transcriptional profile, we conducted a scratch assay on primary mandibular fibrochondrocytes derived from DSRed|NG2 reporter mice. Primary cells extracted from the mandibular condylar cartilage reflect the heterogeneity associated with the cartilage, with only some of the cells expressing NG2/CSPG4 (i.e., DsRed positive). Cell tracing software was used to characterize the distance and velocity of DSRed|NG2-positive and -negative cells during migration. DSRed|NG2-positive cells migrated with a significantly lower distance and velocity than NG2/CSPG4–DsRed-negative cells (Figure 6. D–E; *p* <0.05; *n* = 5/reporter status). This finding illustrates that NG2/CSPG4 expressing cells have an attenuated migratory phenotype, consistent with the knockout data.

**FIGURE 7:**
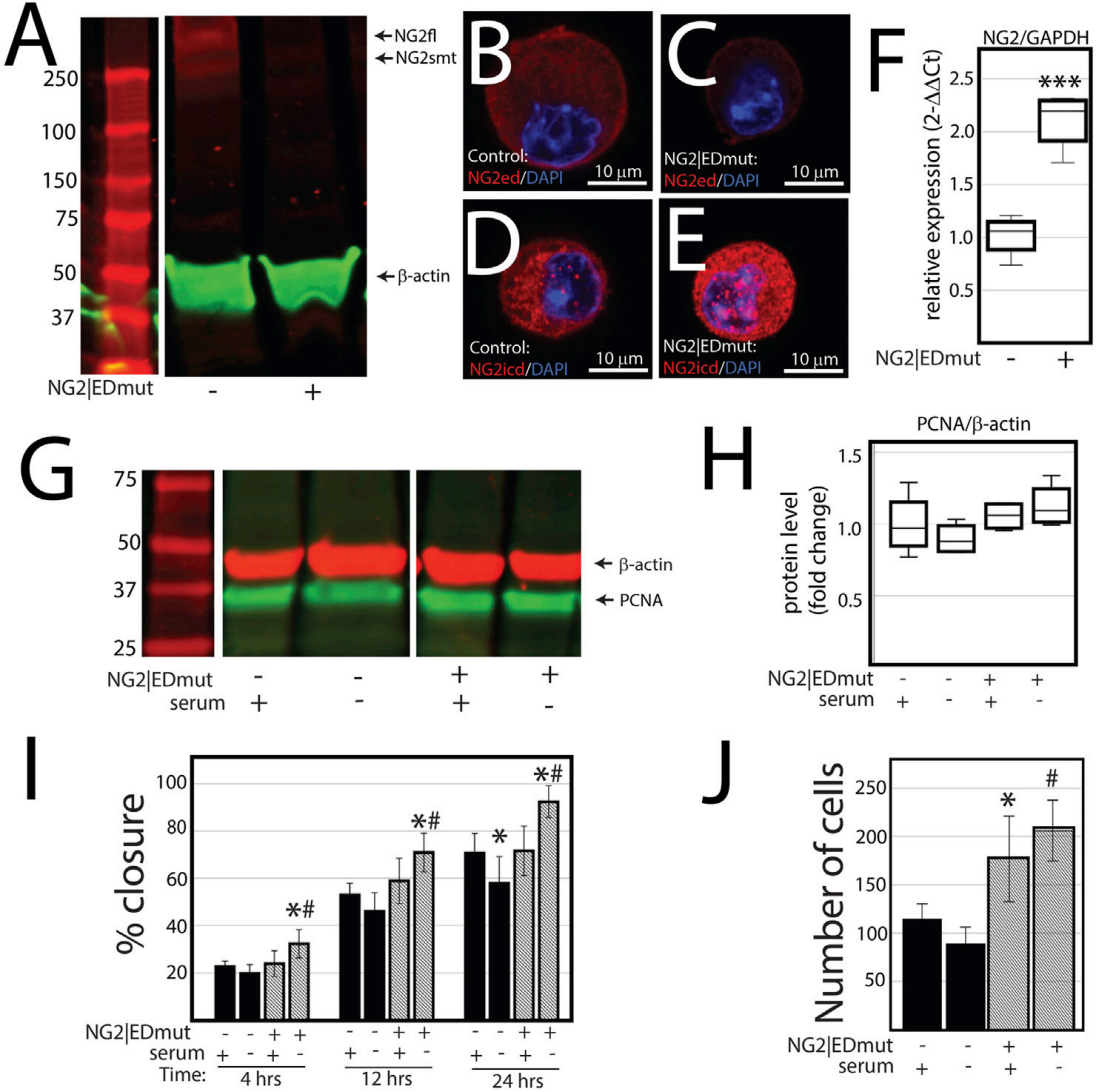
NG2/CSPG4 ectodomain perturbation impacts cell migration. **(A)** Western blot analysis of NG2/CSPG4 of the control and NG2|EDmut cells. These bands are representative of the *n* = 4/experimental group. Continuous Western blots are provided in Supplementary Figure S3. **(B)** Immunocytochemistry from control cells using a polyclonal antibody raise the NG2/CSPG4 ectodomain. **(C)** Immunocytochemistry from NG2|EDmut cells using a polyclonal antibody raise the NG2/CSPG4 ectodomain. **(D)** Immunocytochemistry from control cells using a monoclonal antibody raised the NG2/CSPG4 intracellular domain. **(E)** Immunocytochemistry from NG2|EDmut cells using a monoclonal antibody raised the NG2/CSPG4 intracellular domain. **(F)** RT-qPCR illustrating gene expression changes in NG2/CSPG4 in control and NG2|EDmut cells. **(G)** Western blot analysis of PCNA of the control and NG2|EDmut cells in serum-supplemented and serum-free culture conditions. The bands are representative of *n* = 4/experimental group. Continuous Western blots are provided in Supplementary Figure S3. **(H)** Quantification of PCNA Western blots with values standardized to β-actin and fold change calculated relative to the control–serum-supplemented experimental group. **(I)** Quantification of a migration assay from the control and NG2|EDmut cells in serum-supplemented and serum starvation conditions measured at 4, 12, and 24 h. Data represent the percent closures of the cell-free region time 0. **(J)** Quantification of the number of cells that passed in the cell-free region from the migration assay from serum starvation conditions at 24 h. For all graphs, * represents the comparisons of serum-supplemented and serum starvation conditions with * = *p* <0.05 and *** = *p* <0.001; # represents the comparisons of the control versus NG2|EDmut cell with # = *p* < 0.05.

**FIGURE 8:**
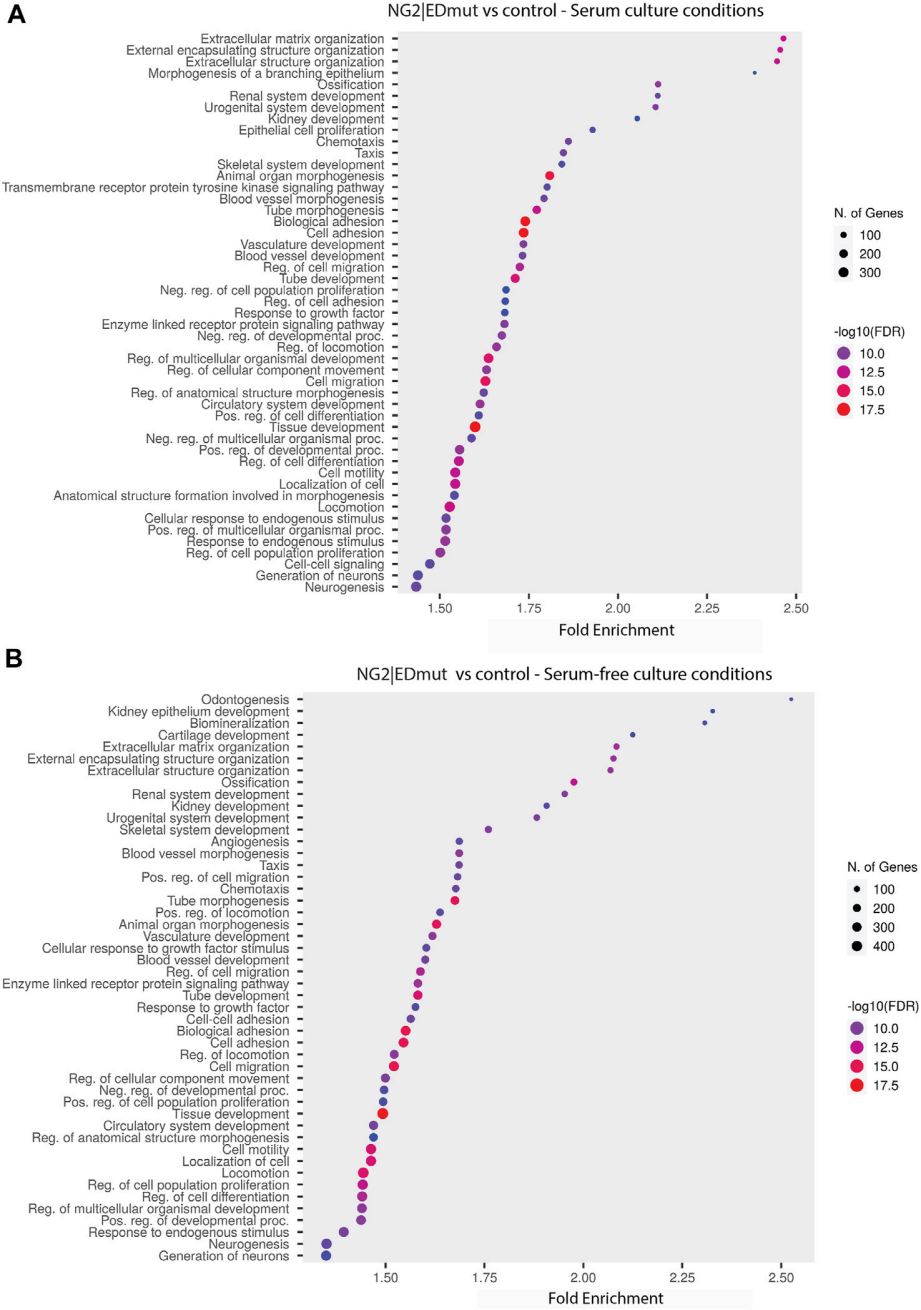
NG2/CSPG4 ectodomain perturbation impacts the transcriptional regulation of cell migration in serum starvation conditions. **(A, B)** Gene Ontology enrichment analysis comparing the differences in biological process terms from a comparison of NG2|EDmut and control immortalized primary mandibular condylar cartilage cells in serum **(A)** and serum-free **(B)** cell culture conditions. Data represent sequencing results from an *n* = 3/genotype per treatment group.

NG2/CSPG4 ectodomain perturbation impacts cell migration. Since the NG2/CSPG4 ectodomain interacts with the extracellular matrix through type VI collagen, we next probed if the NG2/CSPG4 ectodomain was a select and specific regulator of the migratory phenotype of the cell. Mandibular fibrochondrocytes from C57BL/6J mice were immortalized using hTERT and stabilized for five passages. To mutate the ectodomain, the type VI collagen binding region was truncated from the NG2/CSPG4 ectodomain using CRISPR/Cas9 (NG2|EDmut). Western blot analysis of these cells illustrates that the NG2|EDmut lacks robust full length and shed membrane-tethered bands compared to the control (Figure 7A). Immunocytochemistry using a polyclonal antibody against the NG2/CSPG4 ectodomain shows a similar trend, with the control cells having robust membrane-associated NG2/CSPG4 that is greatly diminished in the NG2|EDmut cells (Figures 7B, C). Immunocytochemistry using a monoclonal antibody against the NG2/CSPG4 intracellular domain illustrates robust intracellular/cytosolic NG2/CSPG4 in the control and NG2|EDmut cells (Figures 7D, E). The NG2/CSPG4 intracellular domain could not be resolved on the Western blot in either sample but has been previously validated (Reed et al., 2022). The gene expression of NG2/CSPG4 is significantly higher in the NG2|EDmut cells than in the control (Figure 7F; *p* <0.05; *n* = 4/genotype). There is no statistically significant difference in the rate of proliferation for the transgenic or serum experimental conditions (Figures 7G–H). The rate of migration was significantly different in a genetic and serum-dependent manner. In serum-supplemented culture conditions, the control and NG2|EDmut cells migrate at the same rate. In serum starvation conditions, the NG2|EDmut cells migrate significantly faster than the controls (Figure 7I; *p* <0.05; *n* = 4/genotype) and have significantly more cells migrating into the cell-free regions after 24 h (Figure 7J; *p* <0.05; *n* = 4/genotype). Together, these data illustrate that the NG2/CSPG4 ectodomain is an important regulator of the migratory phenotype of mandibular fibrochondrocytes. The bulk RNA-seq analysis of the NG2|EDmut and control cells in serum supplemented and serum starvation conditions was carried out using Gene Ontology enrichment analysis. Using the biological process ontology set, several significant GO terms associated with cell migration were identified in serum culture conditions including the “Regulation of cell migration” (–log_10_ FDR = 12.5; 200 genes), “Cell migration” (–log_10_ FDR = 15; 300 genes), and “Cell motility” (–log_10_ FDR = 12.5; 300 genes). In serum starvation conditions, significant pathways include “Positive regulation of cell migration” (–log_10_ FDR = 10; 200 genes), “Regulation of cell migration” (–log_10_ FDR = 12.5; 300 genes), “Cell migration” (–log_10_ FDR = 17.5; 400 genes), and “Cell motility” (–log_10_ FDR = 12.5; 400 genes). In both serum and serum starvation conditions, NG2|EDmut cells have a transcriptionally distinct profile affecting genes that regulate cell migration (Figure 8).

## 4 Discussion

This study defines the role of NG2/CSPG4 in the migration of mandibular fibrochondrocytes during normal physiological and cell stress conditions. NG2/CSPG4 has multivalent properties due to the ability to bind the pro-migratory growth factor PDGF and bind with the extracellular matrix through type VI collagen. In serum-supplemented culture conditions, containing the pro-migratory growth factor PDGF, there is no difference in the migration potential of control and NG2/CSPG4 knockout cells. However, pretreatment with mitomycin C resulted in the suppression of cell migration in NG2/CSPG4 knockout cells. In serum starvation conditions, NG2/CSPG4 knockout cells migrated at a higher rate than control cells, irrespective of being pretreated with and without mitomycin C. Using NG2–DsRed reporter cells and cell tracking software, we find that NG2–DsRed-positive cells migrate a shorter distance and at a slower rate than NG2–DsRed-negative cells in serum starvation conditions. When the NG2/CSPG4 ectodomain is perturbed, a similar pattern of migration potential is observed. NG2|EDmut cells migrate faster in serum starvation conditions. Together, these three model systems illustrate that the presence of full-length NG2/CSPG4 on the cell surface attenuates the migratory potential of mandibular fibrochondrocytes.

Our findings from healthy, primary mandibular chondrocytes cultured in serum-supplemented conditions and pretreated with mitomycin C are in agreement with the consensus view from studies using cancer cells. In these cells, NG2/CSPG4 is hypothesized to be a prognostic indicator of metastasis and promotes migration. In chondrosarcoma, siRNA suppression of NG2/CSPG4 attenuated the rate of migration (Jamil et al., 2016). In glia and glioma cells, SP-1 binding to the enhancer region of NG2/CSPG4, and subsequent increase in NG2/CSPG4 protein, increased the migratory potential of the cells (Wilms et al., 2022). A similar pattern is observed in neural cells, with the addition of NG2/CSPG4 antibodies attenuating migration rates (Stegmüller et al., 2002). However, the stage of the cancer can influence the role of NG2/CSPG4 (Hsu et al., 2018), underscoring that NG2/CSPG4 can have different roles in different contexts due to complex ecto- and intracellular domain-mediated processing. In contrast, carrying out the migration assay in serum starvation conditions illustrates that NG2/CSPG4 knockout cells migrate faster than the controls, at a rate more similar to that of the serum-supplemented conditions. This finding underscores that there is potentially an important interaction effect between the transcriptional regulation of the cell and the exposure to cell stress conditions in a high ROS intracellular environment.

During the cell migration assay, cells that migrate into the cell-free area have low levels of cell surface NG2/CSPG4. This could indicate that the primary cells are heterogeneously fated and/or that only cells with low basal levels of NG2/CSPG4 are migrating. This hypothesis is supported by the findings of bulk RNA-seq analysis, illustrating that NG2/CSPG4 knockout cells are transcriptionally distinct with differential gene expression profiles that favor cell migration in both serum-supplemented and serum starvation conditions. However, results from the NG2 reporter line provide a more opaque explanation, with the NG2|DsRed-positive cells migrating slower than the NG2|DsRed-negative cells, but with both cell types moving into the cell-free region. Lower levels of full-length NG2/CSPG4 protein and/or transcript may not necessarily indicate that the protein is non-functional in the molecular mechanics of migration. These cells adjacent to the cell-free region may commit to a migratory phenotype through local endocytic recycling of cell surface NG2/CSPG4 and/or through proteolytic cleavage of the NG2/CSPG4 ectodomain.

NG2/CSPG4 colocalization of β1-integrin at the leading edge of the cell is important and may indicate turnover or recycling of the protein through the lysosomal machinery or proteolytic processing of the ectodomain, as the cell interacts with the endogenous extracellular matrix of the tissue. The endocytosis of NG2/CSPG4 has been implicated in focal adhesion dynamics, with Stonin1 knockout fibroblasts, an NG2 endocytic adapter, associated with the accumulation of cell-surface NG2/CSPG4 and impaired focal adhesion-mediated motility (Feutlinske et al., 2015). The proposed mechanism of action includes the Arf6-integrin disruption of focal adhesions, impacting the directionality of the cells. The capacity for NG2/CSPG4 to regulate migration in melanoma cells was associated with the localization of activated focal adhesion kinases to lipid rafts through the transmembrane cysteine residue (C2230)-mediated assembly of molecular complexes related to syntenin-1 (Yang et al., 2019b). The shedding of the NG2/CSPG4 ectodomain has been illustrated in primary mandibular fibrochondrocytes; however, the molecular functionality of this shedding has yet to be fully resolved (Bagheri Varzaneh et al., 2023). Thus, subcellular localization, and not abundance, could be the most critical parameter regulating migration.

Conversely, high levels of NG2/CSPG4 at cell–cell contacts could indicate contact inhibition of locomotion, where contact with another cell confers polarity and directionality to the cell. This biophysical property is important for regulating the coordinated collective cell migration behaviors such as those in a cell migration assay. In cultured primary mandibular chondrocytes, these data indicate that the abundance of NG2/CSPG4 is dependent on the density of the cells in the plate, confirming previous reports using other cell types (Asher et al., 2005). This result implicates NG2/CSPG4 in the mechanocoupling of cell–cell/matrix junctions and illustrates that this cell–cell/matrix contact is important for retaining cell surface NG2/CSPG4.

The functionality of cell surface abundance may also indicate the need for the cell to interact with the endogenous extracellular matrix produced when confluences is reached, either acting as a cell surface receptor for type VI collagen or as a binding partner with β1-integrins. Changes in collagen VI are one of the characteristics of end-stage temporomandibular osteoarthritis (Reed et al., 2019; Yotsuya et al., 2019). The loss of full-length NG2/CSPG4 or the ectodomain leads to dysfunctional collagen VI matrix adhesion and migration. When collagen VI binding is dysfunctional, sarcoma cells respond with convergent cell survival and adhesion/migration pathways. In other cell types, the targeted deletion of the C-terminal cytoplasmic region also impacts matrix adhesion and cell motility (Fang et al., 1999; Makagiansar et al., 2004; Cattaruzza et al., 2013a). Thus, the proteolytic and endocytic processing of NG2/CSPG4 integrates multiple external stimuli and intracellular signaling networks when shifting toward a migratory phenotype.

NG2/CSPG4 knockout mandibular fibrochondrocytes show significant changes in several key pathways implicated in cell motility and adhesion, including PI3K and MAPK (Reed et al., 2022). NG2/CSPG4 binding with collagen VI promotes the PI3K pathway, regulating cell spreading and motility through bypassing of the canonical integrin transduction mechanisms (Cattaruzza et al., 2013a). The PI3K pathway is associated with cell survival and mTORC1. This could potentially implicate PI3K cell survival as a common signaling pathway regulating migration in serum starvation conditions. PI3K is further implicated in the migratory phenotype of chondrocytes through Runx2 (Fujita et al., 2004). One of the other key cell signaling pathways for chondrocyte migration includes ERK-1/2 (Morales, 2007; Lu et al., 2013). NG2/CSPG4 is necessary for the sustained activation of the ERK cascade (Reed et al., 2022), with ERK differentially phosphorylating the NG2/CSPG4 intracellular domain to regulate proliferation and migration (Makagiansar et al., 2007).

There are several study limitations that were beyond the current scope of this work. Chemotaxis is an important regulator of cell migration, including growth factors such as FGF2 and PDGF. The NG2/CSPG4 ectodomain contains a binding region with high affinity for PDGF-AA, but not PDGF-BB (Nishiyama et al., 1996a; Grako et al., 1999). Future studies will use recombinant PDGF in serum-free conditions to control for the confounding chemotactic effects of PDGF. The NG2/CSPG4 ectodomain also contains chondroitin sulfate chains that can inhibit migration in the cartilage (Davies et al., 2008). Future studies will focus on treatment of cells with chondroitinase ABC for cleaving chondroitin sulfate chains prior to migration. Technical limitations also prevent us from experimentally testing the role of type VI collagen interactions. We attempted to replicate experiments on murine collagen VI-coated plates, but the cells failed to adequately adhere to the plates for the duration of the experiment, particularly so in NG2/CSPG4 knockouts. This finding is consistent with the reports from the literature that NG2/CSPG4 knockout cells have an impaired ability to adhere to type VI collagen. A second technical limitation in the study concerns the use of only two-dimensional culture assays. We replicated the migration experiments using a Boyden chamber, with the serum as the chemotactic gradient, but the primary mandibular fibrochondrocytes failed to migrate through transwell in sufficient quantity to quantify migration.

Despite these limitations, this study has yielded important insights into the functionality of NG2/CSPG4 in cell migration and motility. Future studies on NG2/CSPG4 will broaden our understanding of the migration of stem cells and chondroprogenitor cells as an important part of skeletogenesis. These pro-migratory developmental programs could be utilized for tissue regeneration strategies or improved wound healing outcomes. Tissue engineering approaches would also benefit by the development of homing molecules for stem and/or progenitor cells into the tissue, improving the integration of the engineered scaffold. In the pathophysiology of degenerative arthropathy such as rheumatoid and osteoarthritis, activated fibroblasts like synoviocytes migrate on the articular cartilage and promote cartilage degeneration. The migration of these cells could be targeted to slow the progression of degenerative arthropathy. Together, these findings illustrate that NG2/CSPG4 represents an underappreciated target since it is abundant on the cell surface and contributes to a diverse set of cellular behaviors.

## Data Availability

RNAseq data deposited in the Gene Expression Omnibus: Series GSE245589 https://www.ncbi.nlm.nih.gov/geo/query/acc.cgi?acc=GSE245589. All other datasets are provided in the manuscript and/or Supplementary Material.
