# Supplementary material for: NG2/CSPG4 attenuates motility in mandibular fibrochondrocytes under serum starvation conditions
Source: Front Cell Dev Biol. 2023 Nov 7;11:1240920. doi: 10.3389/fcell.2023.1240920 (PMC10662293; doi:10.3389/fcell.2023.1240920)
Supplement: Supplementary file 4 [file Table2.DOCX]

Supplementary Material

NG2/CSPG4 attenuates motility in mandibular fibrochondrocyte under serum starvation conditions

**Shin Young Ahn^1#^, Mina Bagheri Varzaneh^2#^, Yan Zhao^2^, Jacob Rozynek^2^, Sriram Ravindran^2^, Jonathan Banks^2^, Minahil Chaudry^2^, and David A. Reed^2*^**

*** Correspondence:**David A. Reed
reedd@uic.edu

## Supplementary Figures

## SUPPLEMENTAL FIGURE 01

**
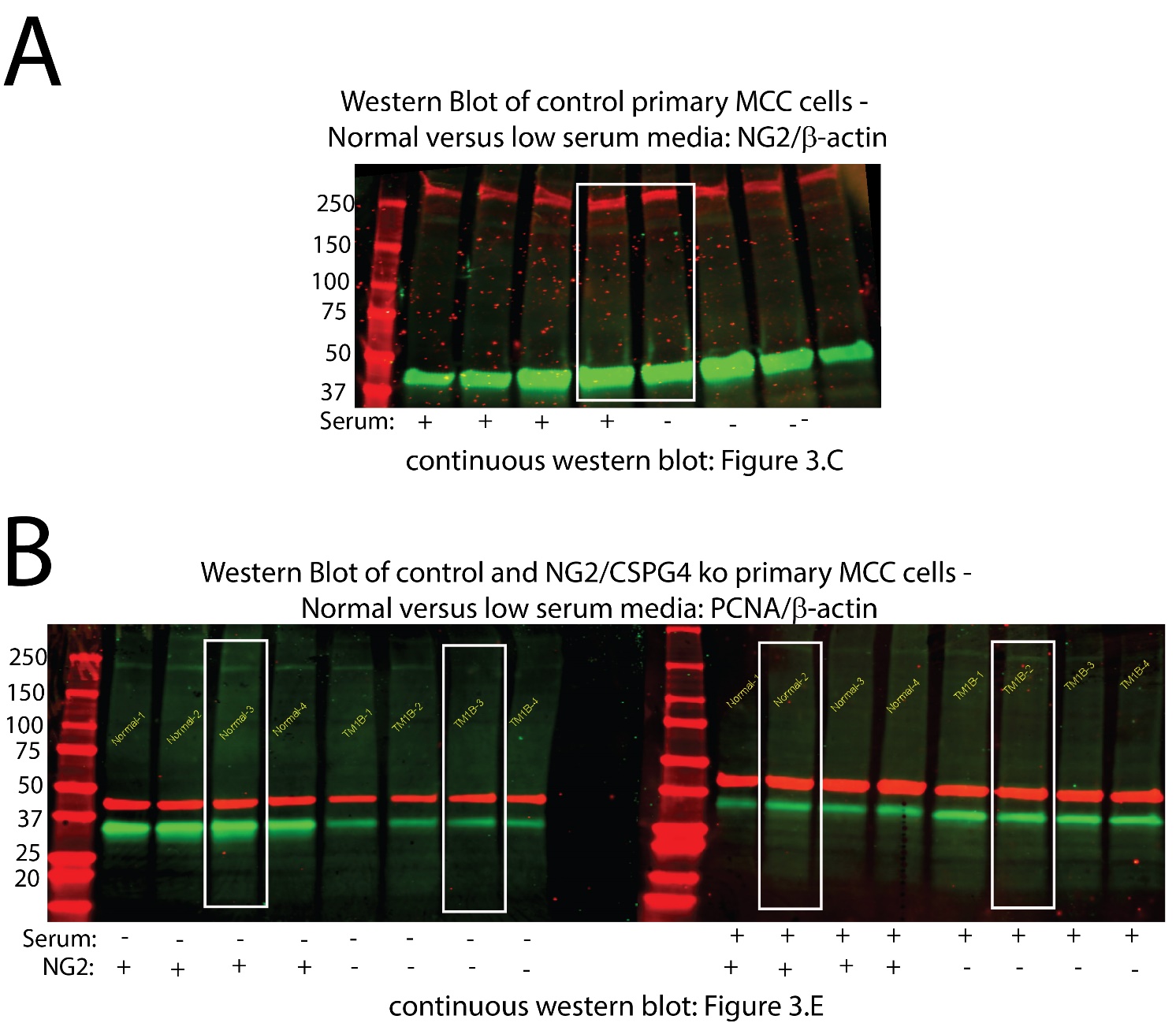
**

**Supplementary Figure 1. (A)** Continuous western blot from Figure 3.C illustrating NG2/CSPG4 and β-actin from primary control mandibular fibrochondrocytes (MCC) in normal and serum starvation conditions. **(B)** Continuous western blot from Figure 3.E illustrating PCNA and β-actin from primary control and NG2/CSPG4 knockout mandibular fibrochondrocytes (MCC) in normal and serum starvation conditions.

**SUPPLEMENTAL FIGURE 02**

**
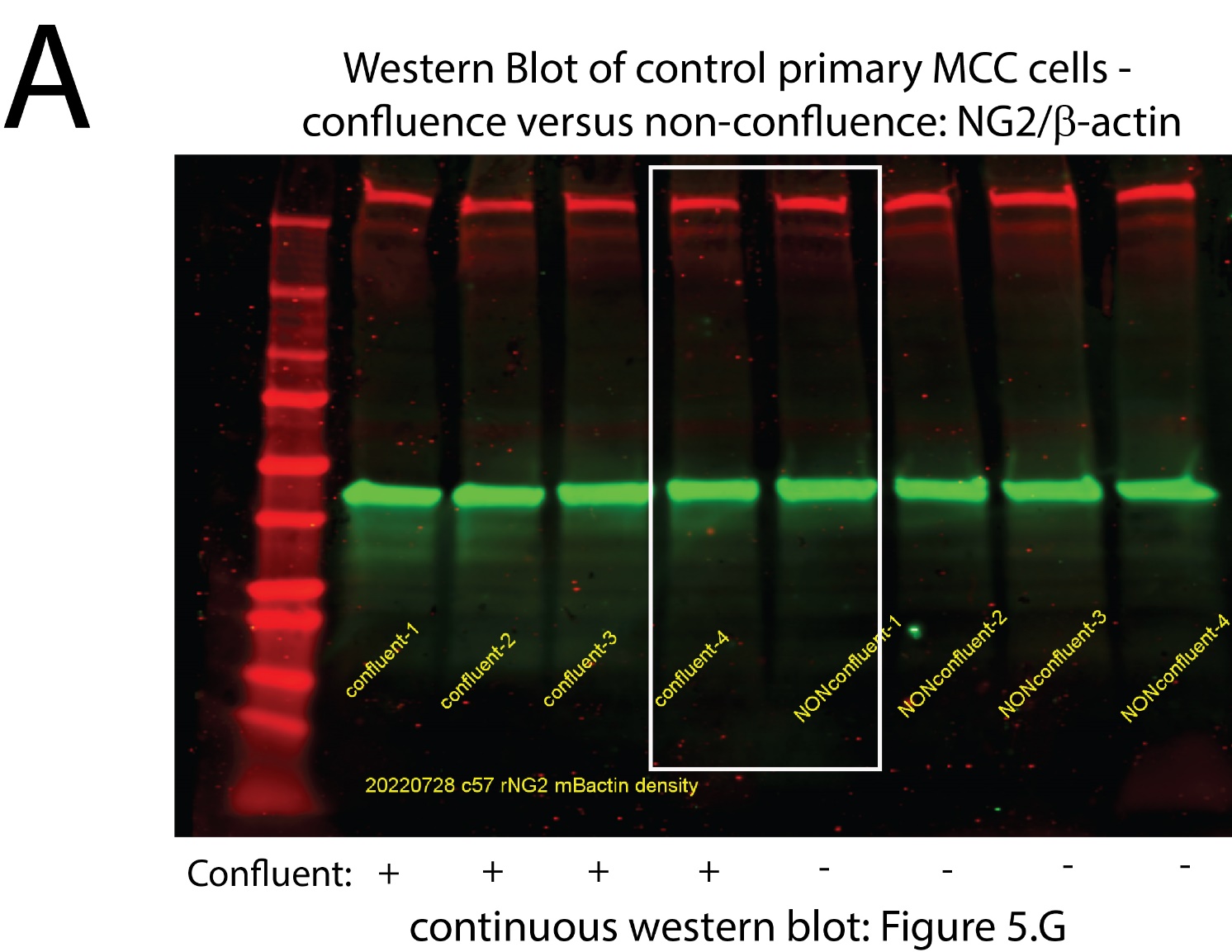
**

**Supplementary Figure 2. (A)** Continuous western blot from Figure 5.G illustrating NG2/CSPG4 and β-actin from primary control mandibular fibrochondrocytes (MCC) in confluent and sub-confluent states

**SUPPLEMENTAL FIGURE 03**

**
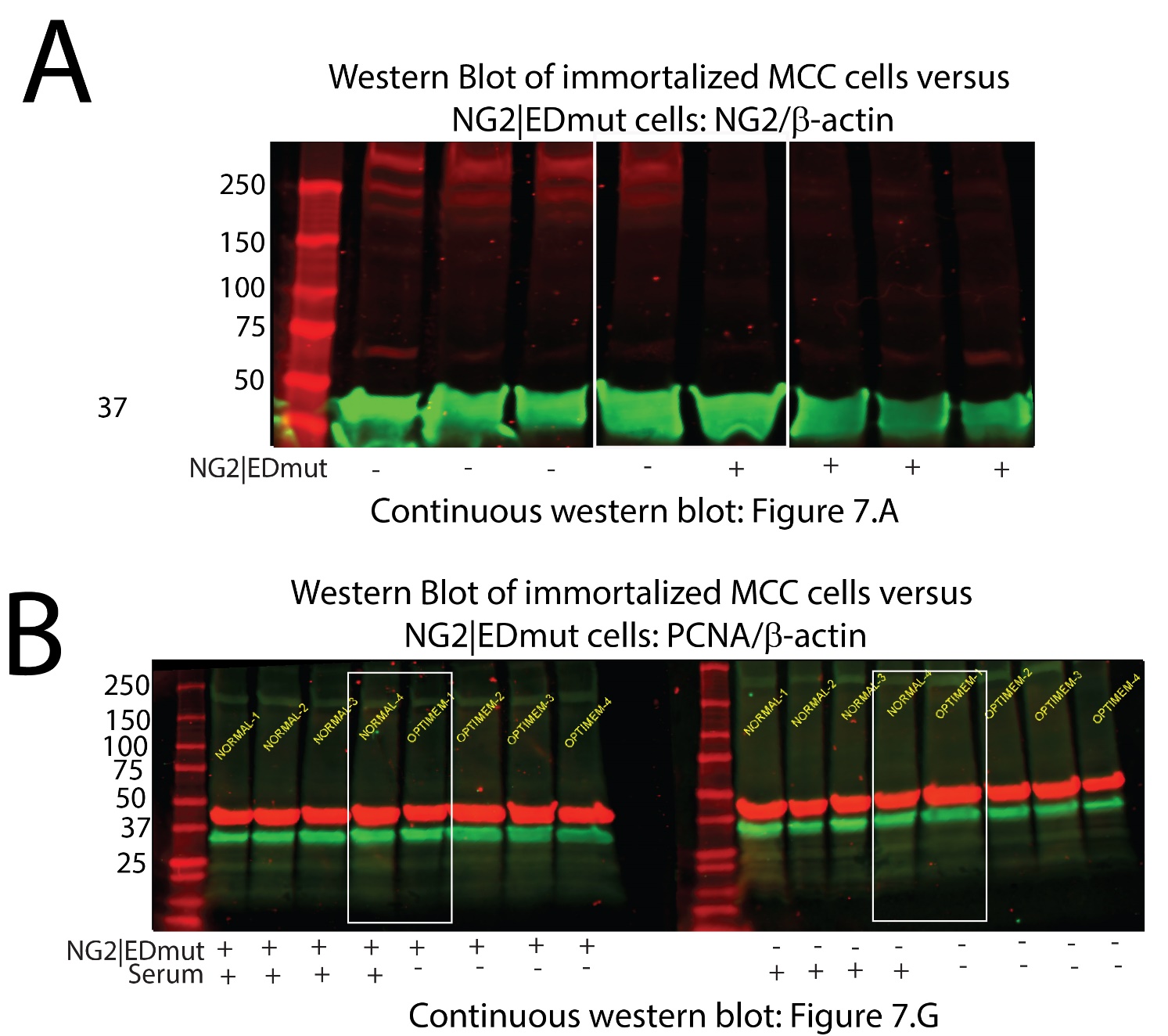
**

**Supplementary Figure 3. (A)** Continuous western blot from Figure 7.A illustrating NG2/CSPG4 and β-actin from immortalized mandibular fibrochondrocytes (MCC) and CRISPR/Cas9 truncated cells (NG2|EDmut). **(B)** Continuous western blot from Figure 7.G illustrating PCNA and β-actin from immortalized mandibular fibrochondrocytes (MCC) and CRISPR/Cas9 truncated cells (NG2|EDmut in normal and low serum conditions

**SUPPLEMENTAL FIGURE 04
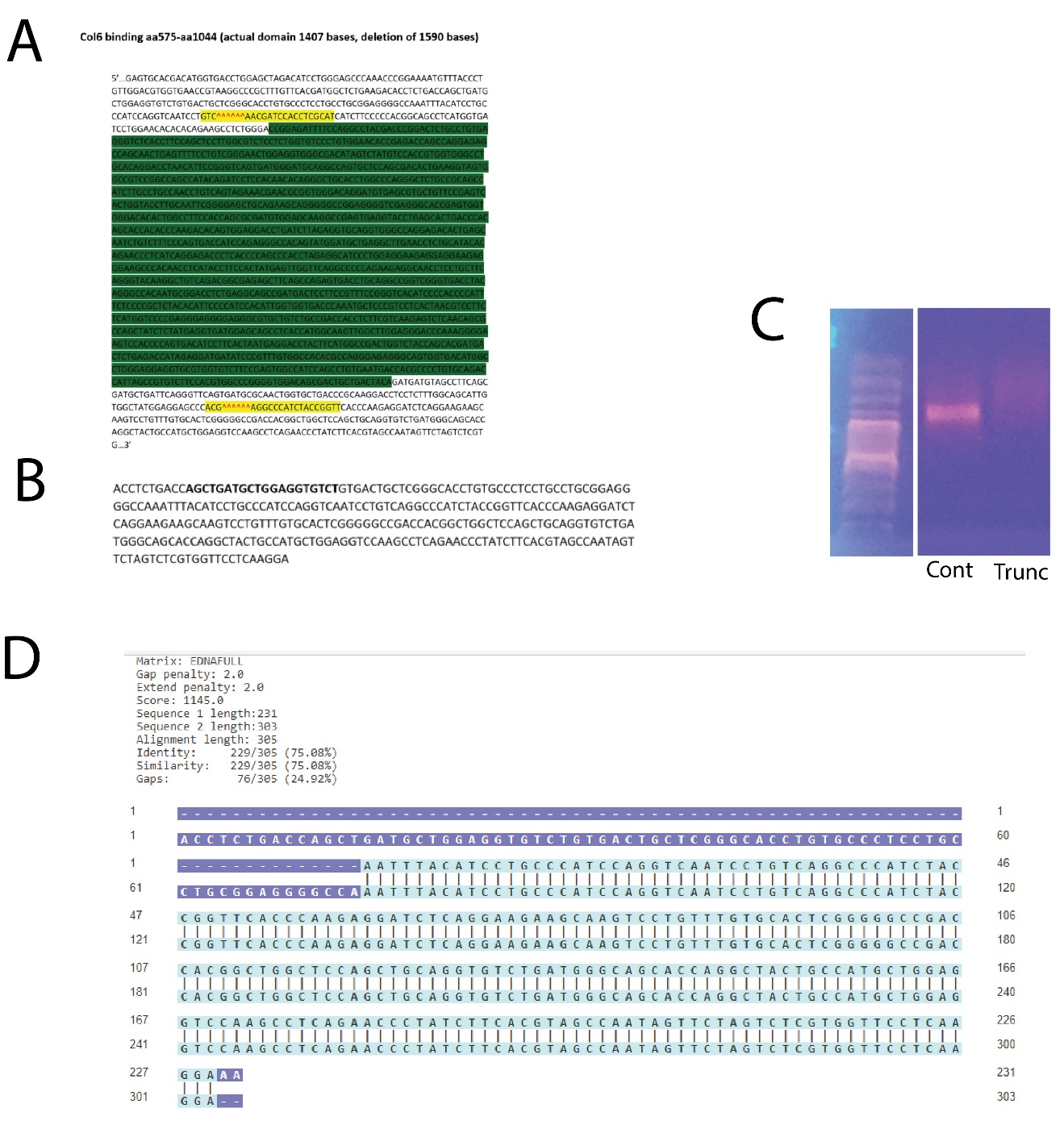
**

**Supplementary Figure 4**. **(A)** Sequence of the type VI collagen binding region on the NG2/CSPG4 ectodomain with targeted Cas9 deletion sites highlighted with red**. (B)** Predicted deletion alignment after truncation. **(C)** PCR results illustrating deletion of the targeted sequence**. (D**) Alignment of the predicted deletion (1) and the actual truncated sequence (2) demonstrating successful truncation of the ectodomain sequence.
